# Novel and Emerging Treatments for Agitation in Schizophrenia and Bipolar Disorder

**DOI:** 10.3390/healthcare13080932

**Published:** 2025-04-18

**Authors:** Sydney A. Mashaw, Ahmed I. Anwar, Judy N. Vu, Austin S. Thomassen, Maya L. Beesley, Sahar Shekoohi, Alan D. Kaye

**Affiliations:** 1 School of Medicine, Louisiana State University Health Sciences Center Shreveport, Shreveport, LA 71103, USA; 2 Department of Psychology, Quinnipiac University, Hamden, CT 06518, USA; 3 College of Natural Sciences and Mathematics, University of Denver, 2199 S University Blvd, Denver, CO 80210, USA; 4 Department of Anesthesiology, Louisiana State University Health Sciences Center Shreveport, Shreveport, LA 71103, USA; 5 Departments of Pharmacology, Toxicology, and Neurosciences, Louisiana State University Health Sciences Center Shreveport, Shreveport, LA 71103, USA

**Keywords:** agitation, schizophrenia, bipolar disorder, dexmedetomidine, olanzapine, patient safety

## Abstract

**Background:** Agitation is a frequent and challenging symptom in schizophrenia and bipolar disorder, characterized by heightened motor activity, emotional distress, and potential aggression. This symptom is most observed during acute episodes, representing a significant burden on patients, caregivers, and healthcare systems. Agitation is a leading cause of emergency department visits and psychiatric hospitalizations, necessitating prompt and effective interventions to ensure safety and mitigate its far-reaching impact. Traditional treatments, including high-potency antipsychotics and benzodiazepines, remain first-line options but are associated with significant drawbacks such as sedation, extrapyramidal symptoms, tolerance, and limited applicability in certain patient populations, especially those with respiratory or cardiac depression and the elderly. Non-pharmacologic strategies like de-escalation techniques and environmental modifications are invaluable but may be impractical in acute care settings, as speed and efficiency are critical in emergent settings. These limitations, including the onset of extrapyramidal symptoms with high-dose antipsychotics and the development of tolerance with benzodiazepines, highlight gaps in care, including the need for faster-acting, safer, and more patient-friendly alternatives that reduce reliance on physical restraints and invasive interventions. **Methods:** This review explores the evolution of treatments for agitation, focusing on alternative and innovative approaches. To highlight these treatments, an extensive review of the literature was conducted utilizing PubMed, Google Scholar, Embase.com, and other search engines. **Results:** Key developments include sublingual dexmedetomidine, recently FDA-approved, which offers sedation without respiratory depression and a non-invasive administration route. Similarly, subcutaneous olanzapine provides a more convenient alternative to intramuscular injections, reducing injection-related complications. Other emerging treatments such as gabapentin, pregabalin, and ketamine show promise in addressing agitation in specific contexts, including comorbid conditions and treatment-resistant cases. A comparative analysis of these therapies highlights their mechanisms of action, clinical evidence, and practical challenges. **Conclusions:** Future directions emphasize intranasal delivery systems, novel pharmacologic agents, and potential roles for cannabinoids in managing agitation. These innovations aim to balance rapid symptom control with improved patient safety and experience. The set back with these emerging techniques is a lack of standardized dosing and protocols. They also face ethical concerns, including the chance of misuse or abuse, as well as regulatory barriers, as they lack FDA approval and their legality changes between states. This review underscores the clinical, practical, and ethical considerations in advancing care for agitated patients, paving the way for more effective and compassionate management strategies in psychiatric settings.

## 1. Introduction

Agitation is a common and often severe symptom experienced by individuals with schizophrenia and bipolar disorder, particularly during acute episodes. Characterized by excessive motor activity, restlessness, and emotional distress, agitation poses significant challenges in clinical settings [1]. The DSM-5 describes agitation as “combative behaviors, particularly in the context of resisting caregiving duties” [2]. Its prevalence in psychiatric populations underscores its clinical significance; agitation is not only distressing for patients but also creates a ripple effect, impacting caregivers, healthcare providers, and the broader healthcare system. In fact, agitation remains one of the leading causes of emergency department visits and psychiatric hospitalizations, emphasizing the need for effective management strategies that ensure safety for both patients and providers [3].

The burden of agitation extends beyond immediate clinical outcomes, affecting quality of life and long-term recovery for patients while placing emotional and financial stress on caregivers. Healthcare providers, meanwhile, face heightened safety risks and resource constraints, especially in emergency settings where rapid and effective intervention is critical [4]. As instances of agitation can swiftly become violent, which can often lead to delay of care, it is necessary to have a well-practiced protocol in place to prioritize patient safety while also maintaining the patient’s autonomy [5]. Despite the availability of established treatment protocols, managing agitation remains fraught with challenges, particularly in acute scenarios requiring swift de-escalation.

First-line treatments for agitation, such as antipsychotics and benzodiazepines, play a pivotal role in stabilizing patients. However, these pharmaceutical options are not without drawbacks. Common side effects, including sedation, extrapyramidal symptoms, and cardiovascular risks, can limit their utility, especially in vulnerable populations. The use of benzodiazepines has even been linked to an increased risk of mortality in cases with schizophrenia, and physicians have been advised to prescribe these medications only in instances of severe need [6]. Additionally, non-pharmacologic interventions—such as de-escalation techniques, behavioral strategies, and environmental modifications—are valuable tools in managing agitation but are not always feasible in high-stakes or resource-limited settings where immediate control of symptoms is paramount [7]. While verbal de-escalation has been found to be successful in patients with agitation, specifically via harboring a strong relationship between the healthcare provider and patient, these methods take a tremendous amount of time and effort, which is not always realistic in an emergent setting [8].

Significant gaps in care persist, highlighting the limitations of current approaches. Many pharmacologic treatments are not rapid acting enough to address acute agitation effectively [9]. Furthermore, some patients cannot tolerate sedation or require alternatives due to medical contraindications [10]. Intravenous or injectable forms of medications, while effective, often necessitate skilled personnel for administration, further complicating care delivery in under-resourced environments. Poor patient compliance and the distress associated with invasive treatments amplify the need for safer, faster-acting, and more patient-friendly options.

Additionally, the management of agitation is not solely shaped by the availability of pharmacologic tools, but also by the broader organizational structure in which care is delivered. For example, a recent investigation into the mental health care delivery system of Italy has highlighted the potential for innovative organizational models that emphasize community-based care and improved coordination between emergency and psychiatric services. By providing both in and outpatient services, as well as emergent services when rendered necessary, they decrease the amount of drastic pharmacologic intervention required. Their findings highlight the role that the service organization plays in optimizing patient outcomes and suggest that without equal improvements at the structural level, even promising pharmacologic advances can fall short [11]. Accessibility, timing, and effectiveness of both first-line pharmacologic treatments for agitation as well as novel approaches discussed in this review depend heavily on systemic and service-level factors, including staffing, provider training, and established care protocols.

The present investigation seeks to provide a comprehensive overview of alternative treatments for agitation in schizophrenia and bipolar disorder, focusing on the evolution of care from traditional approaches to innovative delivery systems. By examining the clinical, practical, and ethical dimensions of emerging treatments, we aim to illuminate pathways for improving outcomes in this challenging aspect of psychiatric care. In this review, we describe mechanisms, benefits, and limitations of novel pharmacologic agents, including sublingual dexmedetomidine, subcutaneous olanzapine, gabapentin, and ketamine. We will also introduce the idea of currently developing treatments, including an intranasal form of olanzapine and the use of cannabinoids. We hope to highlight the importance of personalized treatment strategies in advancing care for agitated patients.

## 2. Historical Treatments for Agitation

### 2.1. Overview of Traditional Approaches

The use of high-potency antipsychotics in the traditional approach to treat agitation includes medications such as haloperidol, and other drugs such as midazolam and ziprasidone [12]. Haloperidol is available in multiple formations, including oral tablets, intramuscular (IM) injections, and intravenous (IV) preparations [13]. There is versatility in the administration routes, which allows for flexibility in different clinical scenarios, such as outpatient maintenance therapy and acute inpatient management for severe agitation. Haloperidol exhibits rapid absorption when administered orally, with the highest plasma concentrations reached relatively quickly [14]. This drug is a dopamine receptor D2 antagonist, which helps mitigate psychotic symptoms and reduce agitation [15].

Haloperidol-mediated affinity for dopamine receptors in the brain allows for efficacy in reducing positive psychotic symptoms and agitation. By blocking these receptors, haloperidol reduces the hyperactivity of dopamine pathways implicated in psychosis. This antagonistic action on dopamine receptors can lead to its therapeutic effects but also leads to its many side effects. Haloperidol, especially in high doses used to treat acute agitation, is considered very toxic, with a high risk of extrapyramidal side effects [16]. In clinical settings, the haloperidol-mediated pharmacodynamic profile necessitates careful dosing to be able to balance its antipsychotic efficacy with risks of adverse effects (Table 1).

Additionally, benzodiazepines are commonly used to manage acute agitation particularly with anxiety or delirium [17]. Persistent agitation can be treated commonly with benzodiazepines and increases the comfort of the patient. There are even cases where a combination of haloperidol and benzodiazepines can be used as an effective management of agitation. Scheduled doses and continuous infusion are also options for administration. Benzodiazepines have a rapid onset and work by inhibiting dopamine neurotransmission through the gamma-aminobutyric acid-enhancing pathway [18]. Benzodiazepines function as possessive allosteric modulators of the GABA-A receptor, which is a ligand-gated chloride ion channel [19]. This binding induces conformational change, and enhances GABA’s effect by increasing chloride ion influx, which hyperpolarizes the neuron and amplifies inhibitor action. This is how benzodiazepines can exert their sedative and anxiolytic effects. Benzodiazepines such as lorazepam are available in oral, intramuscular, and intravenous forms. Lorazepam has a short half-life that makes it suitable for acute management. The setback with benzodiazepines is due to their risk of dependence as well as patient tolerance, which leads to the requirement of higher doses. These higher doses increase the chances of withdrawal and abuse [20].

The combination of sedatives like benzodiazepines with antipsychotics is a common practice with the aim of maximizing therapeutic effects and minimizing the required doses for the drugs. Combining benzodiazepines with haloperidol can potentiate sedation and reduce agitation better than either drug alone [17]. However, there are studies that highlight the insufficient evidence for the benefit of the combination of these drugs [21].

**Table 1:** Traditional approaches to managing agitation.

Drug Type	Mechanism of Action (MOA)	Available Forms	Challenges
Haloperidol (Antipsychotic)	Dopamine D2 Receptor Antagonist	Oral, IM, IV	Extrapyramidal symptoms, neuroleptic malignant syndrome, and dystonia [22]
Lorazepam (Benzodiazepine)	GABA-A receptor agonist	Oral, IM, IV	Tolerance, respiratory depression and dependence [23]
Combination	GABA-A and D2 receptor modulation	Oral, IM, IV	Cumulative side effects, oversedation

### 2.2. Challenges with Older Treatments

The use of high-potency antipsychotics such as haloperidol is associated with significant side effects. Extrapyramidal symptoms (EPSs) are a major concern and potential risk of this treatment, and manifest as acute dystonia and akathisia [22]. These side effects can potentially impact patient quality of life and compliance. The EPS toxicity of haloperidol is very high, which correlates to its challenges as a treatment. There are also risks of neuroleptic malignant syndrome, but they remain very rare in manifestation [24].

Benzodiazepines such as lorazepam also pose challenges and side effects for patients. These side effects include tolerance development, physical development, and physical risk such as sedation, falls, fractures, and cognitive impairment [23,25]. These risks increase in higher doses and in patients with compromised respiratory function [26]. These side effects can impact patient care and the quality of life of the patient. These side effects are especially critical in the elderly population, as cognitive decline can potentiate the impairment experienced on benzodiazepines. The sedative effects can also lead to a reduced ability to perform daily activities.

These traditional treatments also fall short in their ability to address the etiologies of agitation. Treatment-resistant agitation or agitation secondary to mixed psychotic and mood states is difficult to manage with standard antipsychotics or benzodiazepines alone [27]. Traditionally, antipsychotics are used, and more recently, benzodiazepines are used as well. With the use of multiple drugs, the risk of drug interactions increases, which further complicates patient management. There is a massive need for increased customization of treatment depending on patient symptoms and type of agitation.

### 2.3. Need for Advancement and New Therapies

These older treatments of agitation have side effects and limitations and highlight the need for advancements in the management of agitation. The reliance on physical restraints due to the ineffectiveness of traditional medication raises significant ethical concerns and shows the necessity for more effective pharmacological interventions [9]. There is also a critical need for medications that can manage agitation without causing excessive sedation or inducing severe side effects such as EPSs. Developing new drugs with better safety profiles and efficacy and that are tailored to individual patients can reduce overreliance on physical restraints and improve patient care and quality of life. Innovation in pharmacotherapy can offer promising avenues for creating more effective treatments.

## 3. Alternative and Innovative Approaches

Numerous innovative and alternative strategies have been explored for managing agitation in schizophrenia and bipolar disorder, including the use of sublingual dexmedetomidine, subcutaneous olanzapine, gabapentin, pregabalin, and ketamine as novel therapeutic options. Physicians and other healthcare personnel frequently encounter patients experiencing acute agitation. Agitation encompasses a spectrum of motor, emotional, behavioral, and cognitive symptoms and may arise in association with neurological, psychiatric, and general medical conditions.

### 3.1. Sublingual Dexmedetomidine

In a prospective observational study in an urban trauma center that screened more than 43,000 patients, the prevalence of agitation in the emergency department was 2.6% [28]. Although recent guidance for managing agitation emphasizes patient-centered approaches, including verbal and nonverbal de-escalation techniques, pharmacotherapy with sublingual dexmedetomidine has emerged as a promising option due to its efficacy and rapid onset. Dexmedetomidine, an α_2_-adrenergic receptor agonist approved in intravenous form, is available as a sublingual film that bypasses first-pass metabolism through oral absorption, resulting in high bioavailability. In 2022, the U.S. Food and Drug Administration (FDA) approved the use of sublingual dexmedetomidine for the treatment of acute agitation linked to schizophrenia or bipolar I or II disorder in adults. The available evidence supports sublingual dexmedetomidine as a safe and effective treatment, offering rapid onset, minimal need for repeat dosing, and a unique side effect profile that avoids extrapyramidal symptoms and excessive sedation associated with traditional treatments [29]. The most frequently reported adverse effects of sublingual dexmedetomidine are hypotension, bradycardia, and somnolence; however, bradycardia resolved spontaneously without medical intervention, and approximately half of the hypotension cases were deemed clinically insignificant [30].

### 3.2. Olanzapine

Another pharmacotherapeutic option is subcutaneous olanzapine, which has been investigated as an alternative to intramuscular haloperidol. Intramuscular typical neuroleptics are often utilized to manage agitation in schizophrenia when rapid action and compliance are critical. While there are no intramuscular atypical antipsychotics available in the United States, intramuscular olanzapine is being tested because of the favorable profile of oral olanzapine with regard to acute dystonia and QT_c_ prolongation. Unlike haloperidol, a typical antipsychotic that acts predominantly through dopamine D2 receptor antagonism, olanzapine is an atypical antipsychotic with a broader mechanism of action, antagonizing both dopamine D2 and serotonin 5-HT_2A_ receptors, which may contribute to its reduced risk of extrapyramidal symptoms and enhanced efficacy for mood-related symptoms. Olanzapine is a sustained-released intramuscular formulation in which the pamoate salt dissolves slowly after injection into the gluteal muscle, with rapid absorption of the dissociated free base olanzapine into muscle tissue [31].

Subcutaneous olanzapine has thus far been studied primarily in cancer patients with hyperactive or mixed delirium, demonstrating that intramuscular olanzapine is well tolerated when administered subcutaneously, with no injection site toxicity reported after 167 injections in 24 patients [32]. Although the study is limited by the small sample size and the lack of blinding among the nursing staff involved in the experiment, the subcutaneous route may offer safety advantages over intramuscular administration by reducing the risk of injection-related complications while providing comparable efficacy.

### 3.3. Gabapentinoids

Gabapentin and pregabalin, both calcium channel α_2_δ ligands, represent additional therapeutic options that have been explored for their potential to manage agitation and related symptoms in conditions such as bipolar disorder, anxiety, and insomnia. These agents, initially developed for neuropathic pain and seizure disorders, have shown efficacy in addressing heightened emotional and behavioral dysregulation in patients with psychiatric conditions. From a review of 70 studies, three specifically examined gabapentin for the acute treatment of bipolar disorder. Gabapentin was significantly more effective compared to lamotrigine and carbamazepine in decreasing depressive symptoms as measured by the Minnesota Multiphasic Personality Inventory (MMPI-2), though no differences were observed in improvements on the MMPI-2 mania scale. In a crossover study, no statistically significant difference was observed in the Clinical Global Impressions-Bipolar Disorder (CGI-BP) scores during the first phase. However, a long term-study involving 25 patients demonstrated a significant benefit of gabapentin over placebo in CGI-BP change scores, suggesting its potential utility for sustained treatment in bipolar disorder.

For anxiety disorders, gabapentinoids demonstrated significant efficacy compared to placebo across a broad spectrum of anxiety disorders. In generalized anxiety disorder (GAD) and social anxiety disorder (SAD), pregabalin was significantly more effective than placebo, and in conditions such as post-traumatic stress disorder (PTSD), obsessive–compulsive disorder (OCD), and panic disorder (PD), pregabalin also outperformed placebo. However, in participants with alcohol dependence and associated sleep disturbances, gabapentin did not demonstrate improvement in sleep scales compared to placebo, suggesting a potential interaction with alcohol dependence and decreased gabapentin efficacy. Side effects of gabapentin and pregabalin e.g., drowsiness, dry mouth, dizziness, headache, fatigue, and visual disturbances, should be carefully considered when selecting these agents for treatment [33]. In patients with negative symptoms of schizophrenia, including depression and lack of focus, an increase in drowsiness can be detrimental to the treatment of difficult-to-treat negative symptoms [34].

### 3.4. Ketamine

Ketamine, a developing treatment for severe agitation, is an N-methyl-D-aspartate (NMDA) receptor antagonist that modulates glutamatergic neurotransmission and enhances synaptic plasticity. Its traditional use is to induce a state of sedation. Compared to the traditional treatments of severe agitation, such as benzodiazepines or haloperidol, which have a relatively slow onset of action, ketamine has a rapid onset and wide therapeutic index. During a study period, 174 patients were administered sedation for prehospital severe agitation, with 70 of these patients receiving ketamine, and the other 103 patients receiving benzodiazepines. In the results, the ketamine cohort was observed to have a somewhat lower rate of psychiatric evaluation in the emergency department compared to the benzodiazepine cohort [35]. Although ketamine has experienced a resurgence in recent years, clinical practice guidelines advise against its use for procedural sedation in patients with schizophrenia due to the risk of psychiatric exacerbation and decompensation [35]. Ketamine is also associated with an increased risk of dissociation, which could be exacerbated in patients experiencing psychosis [36]. Studies have shown that ketamine’s safety and tolerability profiles are generally favorable at low doses and with very short-term treatment in depressed patients.

Esketamine, an enantiomer of ketamine, could provide a safer alternative to ketamine, as it has a much higher affinity for the NMDA glutamate receptor which allows for a much smaller dose given and decreases the instances of side effects [37]. Its safety and efficacy in treating acute agitation are worthy of exploration, as it may have the benefits of ketamine use without the increased risk of dissociative effects. However, the use of both ketamine and esketamine require extensive exploration, as they still pose a greater risk of dissociation. They should only be administered in clinical settings where patients can be continuously monitored. Future treatment strategies should focus on minimizing the risk of adverse events associated with long-term ketamine use for depression [38].

While numerous pharmacotherapeutic options, including sublingual dexmedetomidine, subcutaneous olanzapine, gabapentin, pregabalin, and ketamine, have shown promise in managing agitation associated with schizophrenia, bipolar disorder, and other psychiatric conditions, further research is needed to optimize their safety, efficacy, and long-term outcomes (Table 2). Sublingual dexmedetomidine provides a non-invasive and relatively well-tolerated option which minimizes respiratory depression; however, its onset is moderate, which limits its use in severe acute agitation [29]. Subcutaneous olanzapine acts more rapidly, making it much more effective for severe agitation. However, it carries the potential for sedation and injection site reactions, although rare. Gabapentinoids are well tolerated and easy to administer orally, but have a very delayed onset of action and are thus better suited for instances of chronic, non-emergent agitation [33]. Finally, ketamine offers the benefit of the quickest onset of action, making it especially useful in dangerous instances of agitation; however, it carries the greatest risk profile, with risks including dissociation which can be catastrophic in patients experiencing psychosis [36]. Overall, in real-world use, sublingual dexmedetomidine and subcutaneous olanzapine work best in controlled, hospital settings with gabapentinoids used in long-term management and ketamine used in only the most serious of emergencies.

## 4. Future Treatments: Emerging Innovations

### 4.1. Intranasal Olanzapine

Intranasal olanzapine represents a groundbreaking advancement in the management of acute agitation associated with schizophrenia and bipolar disorder [40]. This novel delivery system capitalizes on the unique properties of the nasal cavity, which is highly vascularized and offers a direct route to systemic circulation, ensuring rapid onset of action [41]. Additionally, it enables direct access to the central nervous system (CNS) via the olfactory and trigeminal pathways, by passing the blood–brain barrier [42,43,44]. This method overcomes the limitations of conventional drug administration routes, such as oral and parenteral, which struggle to effectively deliver therapeutic agents to the brain [45].

One of the primary benefits of intranasal delivery is its ability to bypass the gastrointestinal tract and hepatic first-pass metabolism. This ensures efficient drug absorption and bioavailability while minimizing systemic side effects [46]. Additionally, utilizing diverse formulations such as nanoparticles, gels, and emulsions can enhance drug solubility, stability, and absorption. These innovations enable controlled release profiles, improving therapeutic effectiveness while enabling lower dose requirements. This improves safety and reduces the risk of overdose [47]. Also, intranasal delivery reduces variability in drug absorption compared to oral formulations, which are influenced by gastrointestinal factors such as motility and food intake. This consistency in absorption ensures more predictable therapeutic outcomes, making intranasal delivery a reliable and precise option [48].

The rapid onset of action of intranasal olanzapine makes it particularly effective in acute scenarios, allowing for the swift de-escalation of agitation episodes and ensuring the safety of both patients and caregivers [49]. Its non-invasive administration enhances practicality, especially in emergency or outpatient settings, where it is preferred over intramuscular (IM) injections to prevent escalation to aggression or violent behavior [50]. Unlike intravenous (IV) or IM injections, intranasal delivery avoids discomfort and reduces procedural risks, improving patient compliance and acceptance [51].

Current clinical data highlight the potential of intranasal olanzapine as an innovative therapeutic option for managing agitation in psychiatric conditions. Two formulations, INP105 (Impel NeuroPharma, Seattle, WA, USA) and NRL-4 (Neurelis, Inc., San Diego, CA, USA), are currently in development, reflecting advancements in this delivery method [40,52,53]. Early-phase trials have shown encouraging results, demonstrating its ability to achieve therapeutic plasma concentrations within minutes, which is critical for rapid symptom control in acute settings. These studies also highlight its favorable safety profile, with mild and transient adverse effects and no significant issues related to respiratory or nasal mucosa irritation [54]. Together, these findings support the potential of intranasal olanzapine as a safe, effective, convenient, non-invasive, and well-tolerated alternative for treating acutely agitated patients by self- or caregiver administration in the home, community, or hospital environments.

### 4.2. Cannabinoids in Managing Agitation

In recent years, cannabinoids, particularly cannabidiol (CBD), have gained attention for their potential use in managing agitation, especially in psychiatric and neurological conditions. CBD, a non-psychoactive compound in cannabis, has shown properties that may serve as both antipsychotic and anxiolytic agents [55,56]. Research suggests that CBD can modulate the endocannabinoid system, influencing serotonin and dopamine receptors, which are crucial in regulating mood, anxiety, and psychotic symptoms. As an antipsychotic, CBD has been shown to reduce symptoms of psychosis in conditions like schizophrenia, likely by reducing dopamine hyperactivity in the brain [57]. As an anxiolytic, it helps alleviate anxiety, potentially by enhancing GABAergic transmission, which has a calming effect on the nervous system [58]. Additionally, cannabinoids may help reduce agitation by addressing common comorbidities like insomnia and pain [59]. These mechanisms collectively support CBD as an innovative approach to managing agitation, which often occurs in the context of heightened psychosis or anxiety in patients with schizophrenia and bipolar disorder [60].

Although the theoretical mechanisms and potential benefits of CBD in managing agitation are promising, the current research landscape is hindered by a shortage of high-quality clinical trials. Most available studies are limited by small sample sizes, heterogeneous patient populations, and variable dosing regimens, leading to inconclusive results [61]. Furthermore, there is a lack of randomized controlled trials directly examining the efficacy of CBD in reducing agitation in cases with schizophrenia and bipolar specifically. This gap in research demonstrates the need for robust, large-scale trials to establish efficacy, clear dosing guidelines, and safety profiles.

Additionally, regulatory hurdles further complicate the integration of CBD into clinical practice. While CBD derived from hemp is legal under the 2018 Farm Bill, it remains subject to strict oversight by the Food and Drug Administration (FDA), which has yet to approve CBD for most medical uses [62]. This regulatory uncertainty is compounded by the lack of standardized guidelines for manufacturing, labeling, and quality control, leading to inconsistencies in product potency and safety [63]. Additionally, as there are limited governmental regulations to CBD and its production, many guidelines change between states. This creates a barrier to clinical application, as patients in some states may have further access to these treatments while others may be restricted. These irregularities in CBD creation and distribution provide the largest blockage to its potentially clinical use.

In addition to regulatory barriers, cannabinoids face a large ethical concern. As regulation is not fixed, the use of the treatments has a potential for misuse and abuse. Some cannabinoids, although not currently CBD, are also associated with the risk of tolerance development, which would not provide a better alternative to current treatments for agitation [64]. These factors create significant barriers to conducting large-scale clinical trials, which are crucial for establishing the safety and efficacy of CBD in treating conditions like agitation associated with schizophrenia and bipolar disorder.

## 5. Discussion

Aggression and agitation in bipolar disorder and schizophrenia are common and necessitate prompt intervention by healthcare professionals [4,65]. Most commonly, agitation symptoms are reported as feelings of unease and nervousness and mostly manifest in mild episodes where patients are often admitted to the hospital [65]. This review provides a comprehensive review of traditional pharmacological interventions utilized for agitation management alongside innovative alternative methods and emerging potential treatments. It emphasizes that addressing the challenges posed by conventional therapies is pivotal to improving patient outcomes and advancing the field of psychiatric pharmacological care.

Conventional treatment methods of antipsychotics and sedatives have long been used in clinical settings to mitigate aggressive symptoms but not without significant side effects, hence the need to look toward novel and alternative treatment forms. Traditional therapies such as haloperidol and benzodiazepine are effective in providing clinical alleviation for aggression. Still, they have significant side effects that are risky and dangerous, including extrapyramidal symptoms (EPSs), which substantially impact patient quality of life and compliance, tolerance issues, and cognitive side effects [22,25]. Another strategy utilized is dual administration of sedatives and antipsychotics, which have provided results such as improved efficacy at lower dosages but carry heightened side effects due to combination drug treatment [17,21].

More promising treatment methods, including dexmedetomidine, subcutaneous olanzapine, gabapentin, and ketamine, offer more favorable side effects when compared to traditional methods alongside more innovative mechanisms of action resulting in more bioavailability. Dexmedetomidine is given as a sublingual film with the ability to bypass first-pass metabolism and olanzapine is administered subcutaneously causing rapid absorption into muscle tissue [29,31]. These pharmacological treatments are slowly advancing and transforming the standard of care, displaying promise with rapid, safe, effective, and minimally invasive relief methods for agitation.

## 6. Conclusions and Future Directions

Further research continues to explore therapy interventions of intranasal olanzapine and cannabinoids. However, these latest advancing treatment options are not without challenges. There is limited availability of high-quality, randomly controlled clinical trials, ethical concerns, and complex regulatory pathways for approval, which can halt the approval process for emerging treatments. Studies focused on real-world application of these treatments are needed, as well as detailed implementation strategies. This is especially true for cannabinoids, which have significant legal and regulatory hurdles required to be permitted in a clinical setting, despite the promise of diminishing aggression and agitation symptoms. The removal of barriers created to stall the development and implementation of new treatment methods requires effort by researchers, clinicians, policymakers, and regulatory bodies to prioritize the continued growth of safer and more effective treatments.

Continued advancement in care and therapies in this field is critical to reduce ethical concerns and adverse side effects for these vulnerable populations. Significant research gaps remain, particularly for newer treatment plans, alongside a lack of understanding and acknowledgment of differences among agitated patients. Recognizing the differing pharmacological effects in men and women with aggression is crucial, as demonstrated within antipsychotic drugs and other pharmacotherapies where women experience more antipsychotic side effects and have faster alleviation of psychotic symptoms at lower doses of drugs compared to men [66]. By prioritizing individualized patient-centered approaches and addressing the ethical and regulatory barriers, healthcare providers and researchers can work together to transform the treatment landscape of aggression commonly seen in bipolar disorder and schizophrenia patients.

## Figures and Tables

**Table 2:** Alternative pharmacotherapy for agitation.

Author	Population	Results	Conclusions
**Study 1: Citrome et al. [30]**	380 participants aged 18–75 years old diagnosed with schizophrenia or schizoaffective disorder. 125 participants received dexmedetomidine 180 μg. 129 participants received dexmedetomidine 120 μg. 126 participants received placebo.	There were no reports of severe or serious adverse effects (AEs). The most common AE was somnolence (180 μg: 23.0%, 120 μg: 21.7%, placebo: 7.9%), hypotension (1.6–6.2%), and dry mouth (4.0–7.8%).	Treatment with sublingual dexmedetomidine 120 μg or 180 μg resulted in greater reduction in agitation compared with placebo.
**Study 2: Smith et al. [29]**	378 participants aged 18–75 years old diagnosed with bipolar disorder. 126 participants received dexmedetomidine 180 μg. 126 participants received dexmedetomidine 120 μg. 126 participants received placebo.	Adverse events were minimal across the study with the most common side effect being somnolence that was rated as mild in many cases.	Sublingual dexmedetomidine is a safe and effective treatment for acute agitation in patients with bipolar I or II disorder.
**Study 3: Wright et al. [39]**	311 participants aged 18 and older diagnosed with schizophrenia. 131 participants received intramuscular olanzapine. 126 participants received intramuscular haloperidol. 54 patients received placebo.	There were significant differences between patients given olanzapine or haloperidol compared with those given placebo 2 h after injection in scores on the excited component of the Positive and Negative Syndrome Scale, Agitated Behavior Scale, and Agitation Evaluation Scale. Significant differences between olanzapine and haloperidol were observed at 15, 30, and 45 min after the first injection.	Olanzapine is superior to haloperidol in reducing agitation 2 h following intramuscular injection and had a significantly more rapid onset of action.
**Study 4: Hong et al. [33]**	Four double-blind, randomized controlled trials were identified. 101 cases were randomized to receive gabapentin, 81 to placebo, 30 to lamotrigine, and 19 to carbamazepine.	Acute treatment showed no clear statistical significance between lamotrigine, gabapentin, and placebo on the Clinical Global Impressions-Bipolar Version change scores (CGI-BP). Long-term treatment with gabapentin showed a significant benefit versus placebo on the CGI-BP change scores.	Caution is indicated when prescribing gabapentinoids as it is not supported by robust evidence except for some anxiety states.
**Study 5: Lebin et al. [35]**	141 EMS patient encounters received either ketamine or benzodiazepine determined by the EMS medical director for those determined to have severe agitation, required sedation, and presented to the ED. Benzodiazepines were administered in 82 visits (58%), and ketamine was administered in 59 visits (42%).	The ketamine cohort was observed to have a somewhat lower rate of psychiatric evaluation in the ED compared to the benzodiazepine cohort (15% vs. 8.6% difference). There was no significant difference in the rate of hospital admission between the ketamine and benzodiazepine cohort, but these were mainly for nonpsychiatric indications.	Administration of prehospital ketamine for severe agitation was not associated with an increase in rate of psychiatric evaluation or psychiatric inpatient admission when compared with benzodiazepine treatment.

## Data Availability

Data sharing is not applicable. No new data were created or analyzed in this study.
